# Culturally appropriate physical activity promotion strategy package among older Chinese adults in the UK: a feasibility randomised control trial protocol

**DOI:** 10.1186/s40814-025-01693-7

**Published:** 2025-11-05

**Authors:** Yang Yang, Kimberly Lazo Green, Nan Zhang, Lisa McGarrigle, Chris Todd

**Affiliations:** 1 School of Health Sciences, Faculty of Biology, Medicine and Health, The University of Manchester, Manchester, M13 9PL UK; 2 Manchester Academic Health Science Centre, Manchester, M13 9NQ UK; 3 Applied Research Collaboration - Greater Manchester (NIHR ARC-GM), National Institute for Health and Care Research, The University of Manchester, Manchester, M13 9NQ UK; 4 Policy Research Unit in Older People and Frailty/Healthy Ageing, School of Health Sciences, Faculty of Biology, Medicine and Health, National Institute for Health and Care Research (NIHR), The University of Manchester, Manchester, M13 9PL UK; 5 Social Statistics, Global Network for Ageing Research On China/Chinese (GNARC), School of Social Sciences, Cathie Marsh Institute (CMI), Manchester China Institute (MCI), The University of Manchester, Manchester, UK; 6 Manchester University NHS Foundation Trust, Manchester, M13 9WL UK

**Keywords:** Physical activity, Older Chinese adults, UK, Feasibility, Randomised control trial

## Abstract

**Background:**

Older Chinese adults in the UK are more likely to be physically inactive and face unique cultural and socioeconomic barriers to physical activity (PA) compared with the general population, yet they lack tailored interventions. Using findings from an evidence review and qualitative interviews, and guided by the Behaviour Change Wheel framework, we developed a culturally appropriate PA promotion package to enhance the capability, opportunity, and motivation of older Chinese adults in the UK to engage in PA.

**Aim:**

This study aims to assess the feasibility and acceptability of conducting a randomised controlled trial (RCT) of culturally appropriate PA promotion strategies among older Chinese adults in the UK.

**Methods/design:**

This is a two-arm pragmatic feasibility RCT. Community-dwelling individuals who self-identify as Chinese, reside in the UK, are aged 60 years and older, and do not meet the World Health Organization (WHO) PA recommendations (i.e. < 150 min moderate PA or balance/strength exercise < 2 times per week) will be recruited. Participants will be randomised to receive either the culturally appropriate PA promotion strategy package or the WHO PA recommendation leaflet. Outcome data will be collected at baseline, 12 weeks, and 18 weeks post-randomisation. Primary outcomes will include feasibility measures (e.g. recruitment, compliance, adherence, and follow-up rates), intervention acceptability (qualitative interviews), and both subjective PA levels (PA Scale for the Elderly) and objective PA levels (Realalt 3DTriSport 3D Pedometer). Secondary outcomes will include the Brief COM-B (Capability, Opportunity, Motivation—Behaviour) questionnaire, physical function assessments (Short Physical Performance Battery, SPPB), and quality of life (Control, Autonomy, Self-realisation, and Pleasure-12,CASP-12). The feasibility of the study will be evaluated based on the pre-specified progression criteria.

**Discussion:**

This is the first study to assess the feasibility and acceptability of conducting an RCT of a culturally appropriate PA promotion strategy package among older Chinese adults in the UK. The findings from this study will inform improvements in PA promotion strategies and guide the development of a full-scale RCT, with the ultimate goal of creating culturally appropriate, sustainable interventions to improve the health and well-being of older Chinese adults in the UK.

**Trial registration:**

The study protocol was registered on the Open Science Framework on 19 November 2024 (https://doi.org/10.17605/OSF.IO/XQHJS).

**Supplementary Information:**

The online version contains supplementary material available at 10.1186/s40814-025-01693-7.

## Introduction

The Chinese population is one of the largest ethnic minority groups in the UK [1]. According to the most recent census data from 2021, the Chinese ethnic group in England and Wales comprised 445,646 people, accounting for 0.7% of the total population. While a majority of individuals from Chinese ethnic groups are of working age (16 to 64 years), there is still a notable percentage (6%) aged 60 and above [2], including those who migrated at a younger age and grew old in the UK, as well as those who migrated later in life to join their migrant families. In this paper, we use the term “older Chinese adults” to be consistent with academic writing practice. However, when interacting with our participants, we use the phrase “Chinese seniors” (“长者”) in line with community preferences as suggested by our public and community involvement and engagement (PCIE) advisors.

The well-being of older adults from ethnic minority groups has gained increased attention from researchers and policymakers [3]. Compared with the native population, older Chinese adults in the UK face more health problems and adopt less healthy lifestyles due to additional cultural, economic, and socioeconomic challenges [4]. For example, older Chinese adults in the UK are more likely to be physically inactive compared to their native counterparts, despite the well-documented benefits of physical activity (PA) [5, 6]. According to Ethnicity Facts and Figures, in the 55–74 age group, the Chinese ethnic group had a lower proportion classified as “physically active” compared to both the White British population and the national average [7]. On one hand, the low language proficiency and cultural barriers diminish their willingness, opportunity, and capability to participate in local PA programmes. On the other hand, compared to the native population, there are fewer resources and services specifically tailored to this ethnic minority group [5].

PA promotion interventions have been tested to increase the engagement of older adults in PA [8]. We conducted a systematic review of evidence on the use of PA promotion interventions for older adults from the Chinese diaspora and found that no intervention has been developed specifically for older Chinese adults in the UK. This highlights the importance of identifying culturally appropriate PA promotion strategies for this population. We also held a series of qualitative interviews with older Chinese adults in the UK, and found that factors influencing their PA behaviour are related to capability, opportunity, and motivation, as outlined in the COM-B (Capability, Opportunity, Motivation-Behaviour) framework [9], (Yang Y, Zhang N, McGarrigle L, Lazo Green K, Todd C: Culturally appropriate physical activity promotion strategy package among older Chinese adults in the UK: a feasibility randomised control trial protocol, Unpublished).

Based on findings from a systematic review and interviews with the target population, supported by PCIE activities, we identified a series of Behaviour Change Techniques (BCTs) to enhance the capability, opportunity, and motivation of older Chinese adults to engage more effectively in PA. These strategies are presented as a three-part package, comprising culturally appropriate PA booklets, three monthly workshops, and a virtual social media support group.

Since this is the first PA promotion package specifically designed for older Chinese adults in the UK, this study seeks to conduct a feasibility randomised controlled trial (RCT) to evaluate key study procedures—such as recruitment, randomisation, attrition, data collection, data analysis, and outcome measure suitability. Additionally, this study will include qualitative interviews with participants to assess the acceptability of the culturally appropriate PA promotion strategy package.

This study will answer the following research questions:

Study preparation stage

- What is the willingness among older Chinese adults in the UK to participate in this study?
- What is the willingness among older Chinese adults in the UK to undergo randomisation?

For older Chinese adults who participate in the study

- Are participants willing to remain in the study for its entire duration?
- Are they willing to engage in PA promotion strategies, including attending workshops, using the logbook, wearing the pedometer and joining the social media group?
- What are their perceptions of the PA promotion strategies?

Data collection and analysis

- Are they willing to participate in assessments and complete the data collection at the three time points?
- Are the outcome measures appropriate and acceptable for older Chinese adults in the UK?

Effectiveness questions

- How do older Chinese adults’ subjective and objective PA, physical function, and quality of life change after the intervention in both the intervention and control groups?

## Methods

### Study design

This is a two-arm pragmatic feasibility RCT study with an exploratory design framework. Alongside the trial, qualitative interviews will be conducted to evaluate the acceptability of the PA promotion strategy package, as well as the trial procedures, from the perspective of older Chinese adults in the UK. We report the protocol of the feasibility RCT following the CONSORT 2010 statement for randomised pilot and feasibility trials [10]. The study protocol has been registered in the Open Science Framework (10.17605/OSF.IO/XQHJS).

### Study setting and participants

#### Study setting

This feasibility study will be conducted in the community setting of Manchester, UK, which is known for having a substantial Chinese population. While the Chinese diaspora is geographically dispersed throughout the UK, Manchester stands out as one of the regions with the largest Chinese population, with approximately 3.0% (12,644) of all Chinese individuals residing there [11].

#### Eligibility criteria for participants

We will recruit participants who meet the following inclusion criteria:

- Adults aged 60 years or above;
- Self-identified as Chinese ethnicity;
- Speaking either Cantonese, Mandarin, and/or English;
- Able to read and write Chinese or English;
- No time requirement for their stay in the UK, but they should have settled in the UK as residents and not as tourists.
- Self-report not meeting the PA guidelines for older adults (engaging in less than 150 min of moderate-level PA each week and performing balance, strength, and flexibility exercises less than twice a week);
- Self-report being able to walk around at home independently;
- Can provide informed consent.

We however will exclude:

- Those who self-report that they already meet the PA guidelines (engaging in more than 150 min of moderate-level PA each week and performing balance, strength, and flexibility exercises more than twice a week), as this study focuses on older Chinese adults with insufficient PA.
- Those who plan to be away from Manchester for more than 2 months during the intervention period.
- Participants with diseases where exercise is contraindicated will be excluded to ensure the safety of the participants (see Table 1). A self-reported approach will be used to identify these conditions, including cognitive impairment, as it is considered more acceptable by PCIE participants than formal assessments such as the MMSE (Mini-Mental State Examination), which were viewed as burdensome. To mitigate potential bias, the research team will monitor participants throughout the study. Participants who show signs of impairment after giving consent, such as difficulty completing data collection or following instructions, will be excluded.

**Table 1:** List of conditions contraindicating exercise participation

Participants who self-report any of the following diseases will be excluded:
o Uncontrolled pain;
o Uncontrolled angina;
o Major heart surgery in the last 6 months
o Resting SBP > 180 mmHg or resting DBP > 100 mmHg
o Tachycardia > 100 bpm
o Acute systemic illness (cancer, pneumonia)
o Visual or vestibular disturbances
o Significant drop in BP during exercise
o Unstable or acute heart failure
o Unable to maintain seated upright posture due to neurological deficits
o Recent injurious fall without a medical examination
o Serious impaired cognition (to the extent of being unable to follow simple movement instructions)
o Any other clinical condition that the person’s GP or clinician considers would make them unsuitable for participation in an exercise programme

### Recruitment, consent and screening

Participants will be recruited through different approaches: (1) Participants will be approached and recruited through the support of community organisations in Manchester. (2) Participants who have already taken part in the first stage of this project through interviews and have given explicit consent to be contacted for future studies will be contacted to inform them about the intervention study. (3) Recruitment advertisements will be posted in Chinese community centres and Chinese-speaking churches. (4) Advertising will also take place through study-specific social media accounts and platforms such as the Chinese social media app WeChat. Participants will also be recruited through word-of-mouth approaches.

Any interested participants can contact the researcher directly and will be provided a copy of the participant information sheet (PIS). Potential participants will be given at least 24 h to decide whether or not to take part. Potential participants who are interested in the study will be asked to provide written consent (Appendix 1). After signing the consent form, participants will be asked to answer a series of screening questions to determine their eligibility. Once eligibility is confirmed and interest is reaffirmed, baseline data will be collected. A gift voucher will be provided to participants who complete the assessment, which aims to improve recruitment rates and reduce dropout rates. A convenience sampling approach will be utilised. We will strive to recruit participants from a broad spectrum of demographics, including gender, age, PA level, and language backgrounds, encompassing speakers of Mandarin and Cantonese.

### Sample size

The determination of the sample size is driven by practical considerations and the need to obtain meaningful insights into the feasibility aspects of the larger trial, which is different from estimating effects or hypothesis testing in an RCT that requires robust sample calculation. There is limited guidance on determining the sample size for feasibility trials. A recent review shows that the median target sample size of pilot and feasibility studies was 30 (with an interquartile range of 20–50) per arm [12]. Julious (2005) proposed a rule of thumb of 12 participants per group [13].

Considering the timeline of the project, it is essential to balance sample size requirements with time constraints.

We aim to include at least 12 participants per study arm, totalling 24 participants. To account for an anticipated 20% dropout rate, we will recruit at least 30 participants. Assuming a retention rate of 80% with a sample size of 30, the 95% confidence interval for the number of retained participants is approximately 19 to 27.

This study targets older Chinese adults in the UK with insufficient PA. Chinese as an ethnic minority group in the UK is often considered a hard-to-reach group for research. Based on insights from the PCIE presentations, recruiting physically inactive Chinese adults presents even greater challenges, as those who are used to sedentary lifestyles are reluctant to participate in any activities, including research.

### Randomisation

Randomisation is the process of assigning participants to different groups to ensure an unbiased and equal distribution of characteristics among them [14]. After completing baseline measurements, participants will be randomised into control or intervention groups using sealedenvelope.com [15] block randomisation with randomly selected blocks of 2 or 4 or 6. Given the small sample size, randomisation will be stratified by gender to prevent type I error and improve power for small trials [16]. Gender was selected based on the suggestions from the previous interviews with older Chinese adults and PCIE participants, as it influences the PA behaviour of older Chinese adults. Upon completing the baseline data collection, the researcher will contact the university research support (NZ) to log in to the sealed envelope for the assignment information. The researcher will then report the participant’s unique ID and gender to complete the randomisation. Research support will only be involved in the random number generation and allocation process and will not be involved in the recruitment procedure.

**Fig. 1:**
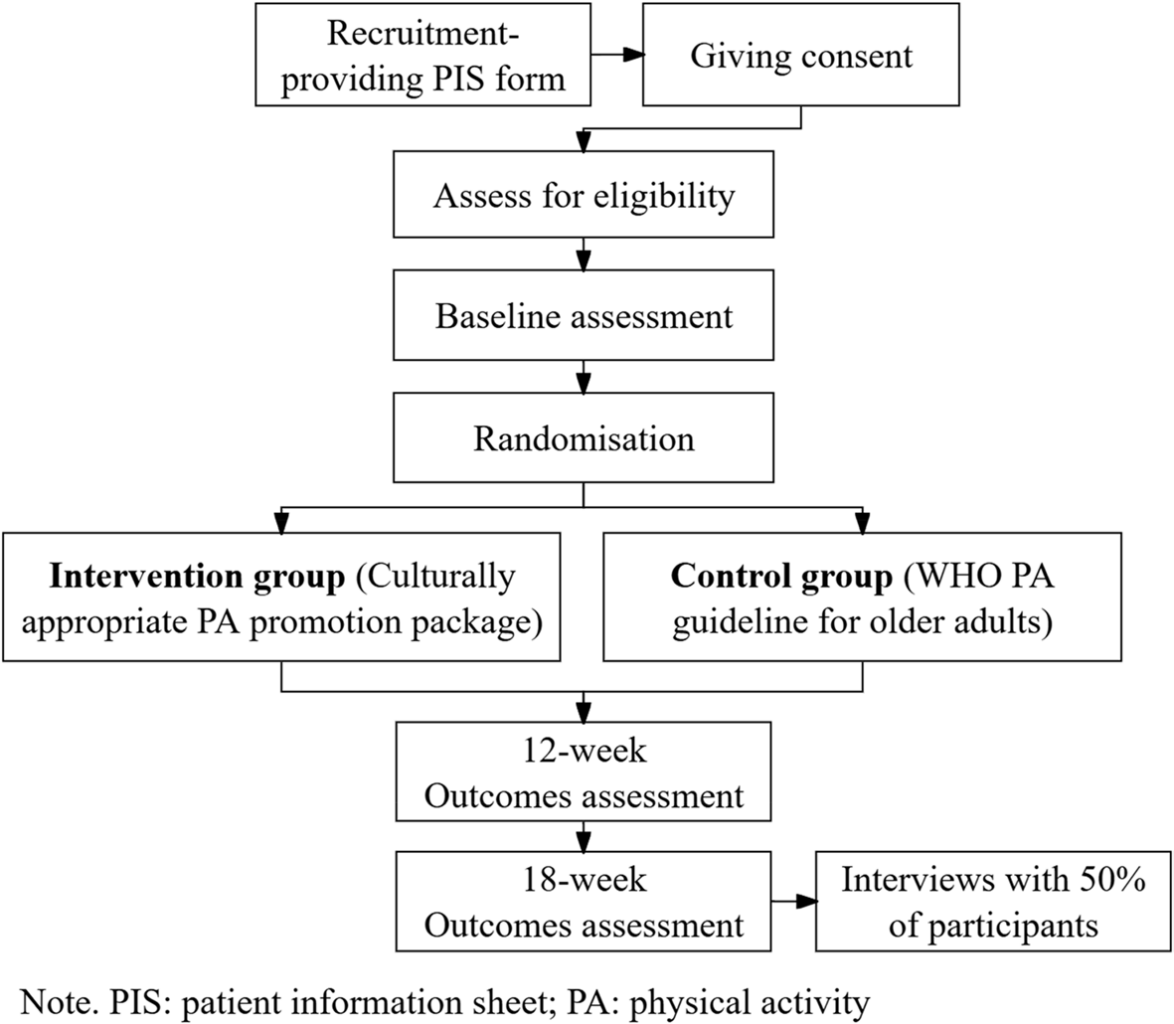
Flow of participants through the study

The flow chart in Fig. 1 summarises the randomisation, intervention, and data collection process.

### Blinding

Blinding refers to the process of concealing the allocation of participants to different study arms from the participants and/or the researchers involved in the study to ensure the validity of the study results [17]. However, considering the PA promotion strategies are “active” interventions, which will be delivered by the leader researcher, therefore, it is not possible to blind either the participants or the researcher.

The baseline assessment will be conducted before randomisation, ensuring the data collection occurs before any interventions are introduced. Subsequently, outcome assessments will be conducted by the lead researcher, with support from a research assistant who is unaware of the randomised group assignments. This approach is intended to minimise bias in the outcome assessment process and enhance the validity of the study’s findings. The lead researcher will carry out the data analysis. Although the “blinding” strategies cannot be employed in the trial due to the nature of the PA promotion intervention, we will strive to minimise biases through clear instructions, standardised procedures, and transparent reporting of blinding procedures to enhance the validity and reliability of the study findings.

### Intervention group

Participants randomised into the intervention group will receive the culturally appropriate PA promotion package. The strategies in the package were developed based on 27 BCTs and will be delivered through three parts: a series of booklets, three workshops, and a virtual social media support group. The intervention content is presented in Table 2.

**Table 2:** The intervention content table

Component	COM-B	Intervention functions	Behaviour change techniques	Intervention description	Evidence from the qualitative interview with the target population
**Booklet**					
**Booklet- page 1**	**Capability**	Education Persuasion	5.1. Information about health consequences 5.3. Information about social and environmental consequences 5.6. Information about emotional consequences	A page introduces the health, social, and emotional benefits of PA and encourages participants to identify the benefits most relevant to their personal needs	“*I wonder if physical activity is really that important. The older lady who lives next door is 94 years old, she never exercises but is very healthy. So, is PA really that important?”*
	**Motivation**				
** Booklet-page 2**	**Capability **	Education	9.1. Credible source	A page provides information about the WHO PA recommendations for older adults	*“I don’t know how much PA I should do; I believe older adults should only walk slowly….”* *I think most older adults pay little attention to the guidelines…*
	**Motivation**				
** Booklet-page 3**	**Motivation **	Education Modelling	1.1. Goal setting (behaviour) 1.2. Problem-solving	A page to help participants identify the gap between their current PA level and the recommended PA guidelines A page guides participants in identifying the barriers they face in attending PA	*“I can check how many steps I’ve taken on my phone, so I set a target, like 10,000 steps each day. Then I walk with the purpose of achieving my target. I think this motivates me”* *Chinese seniors in the UK are a diverse group and face various barriers when attending PA*
	**COM-B problems identified**				
** Booklet-page 4**	**Opportunity**	Education Enablement	1.2. Problem-solving 8.3. Habit formation	A page offers tips on how to integrate PA into daily life	“*Physical activity is not really part of my routine. I have to take care of my grandchildren, so how can I find time to exercise?”*
	**Capability**				
** Booklet-page 5**	**Capability**	Education	4.1. Instruction on how to perform the behaviour	A page introduces safety tips for older adults participating in PA	*“PA may be dangerous for our older adults. Many older adults, like me have high blood pressure, and it’s dangerous when my heartbeat increases”* “*Falling is my biggest concern* *My husband passed away two weeks in the hospital after he fell in the garden. I believe safety is the most important thing.”*
	**Motivation**				
** Booklet—page 6**	**Motivation**	Education Modelling	5.1. Information about health consequences 5.3. Information about social and environmental consequences 6.2. Social comparison 16.3 Vicarious consequences	A page provides examples/stories of how PA has benefited other Chinese seniors in the UK in terms of health, social connections, and emotional well-being	“*What’s the point? I used to be very active, but my husband died two years ago. I just don’t have interest in anything, including physical activity*” *“I’ve worked very hard my whole life, and now I’ve just become a bit lazy, you know? I just want to take it easy, like watching TV or playing mahjong with my friends.”*
** Booklet—page 7**	**Opportunity**	Enablement	3.1. Social support (unspecified)	A page offers guidance on how to find additional support, such as from family members, peer support and the health care system	“*I’m not really sure what type of exercise suits me, and the GP usually doesn’t discuss exercise with me. You know, the appointment time is very limited*.” *“I live by myself, and my grandson calls me to remind me to exercise every morning… so I just walk around inside my house.”*
** Booklet—page 8**	**Opportunity**	Enablement	3.1. Social support (unspecified) 7.1. Prompts/cues	A page provides information about existing local Chinese PA programmes	“*I just moved to the UK. I enjoyed exercising when I was in Hong Kong, but I don’t know about the exercise programs here. If I knew, I would love to join*.”
** Additional booklet-** Otago Exercise Booklet	**Capability**	Training	4.1. Instruction on how to perform the behaviour 9.1 Credible source	The Chinese version of Otago Home Strength and Balance Booklet	“*I only walk, and I walk at least half an hour a day. I don’t know how to do strength and balance exercises.”* *“Sometimes, I follow exercise videos on my phone, although I’m not sure if I’m doing them correctly*.”
** Additional booklet –** PA Logbook	**Motivation**	Enablement	1.1. Goal setting (behaviour) 1.4. Action planning 1.5 Review behaviour goal(s) 1.6 Discrepancy between current behaviour and goal 2.3. Self-monitoring of behaviour 2.5. Self-monitoring of outcome(s) of behaviour 5.4. Monitoring of emotional consequences	Provide a PA logbook for setting targets, monitoring PA behaviour, and tracking achievement, such as improvements in balance, strength, and emotional well-being	“*I was very busy and worked hard when I was young, and now that I’m retired, I prefer quiet activities, like watching TV. I enjoy my life, but physical activity has never been a part of it*.”
	**Capability**				
**Workshop**					
** Presentation**	**Capability**	Education Persuasion	1.2. Problem-solving 5.1. Information about health consequences 5.3. Information about social and environmental consequences 15.1. Verbal persuasion about capability	The researcher will give a short presentation during each workshop to introduce the information in the booklet, helping participants better understand the content	“*You know, some older adults may not enjoy reading, and some of us have poor eyesight or low literacy level. So they may not be able to read…”* *“I think gathering people together for a lecture would be more effective, and it would also provide an opportunity for people to meet each other.”*
	**Motivation**				
** Group discussion**	**Motivation**	Persuasion Modelling	3.1. Social support (unspecified) 6.2. Social comparison 12.2 Restructuring the social environment	Participants will be able to discuss their physical activity during each workshop and share their PA experiences	“*I think gathering together like today to talk about PA is very interesting. When I hear about others doing so much exercise, I feel like I also need to exercise. You know, I want to be healthy too”*
** Exercise demonstration**	**Capability**	Training	2.2. Feedback on behaviour 4.1. Instruction on how to perform the behaviour 6.1. Demonstration of the behaviour 8.1. Behavioural practice and rehearsal 9.1 Credible source	A Chinese-speaking physiotherapist will be invited to demonstrate how to perform the Otago strength and balance exercises	“*You know, if you can invite a professional to show us how to exercise, we would be more likely to believe it. The most important thing is to design something that is really reliable, so we can trust it. Otherwise, people may not listen to you*”
**Social media support group**					
** Encourage/remind message from the researcher**	**Motivation**	Persuasion Incentivisation	3.1. Social support (unspecified) 3.2. Social support (practical) 3.3. Social support (emotional) 7.1 Prompts/cues 10.4. Social reward	The researcher will send messages to encourage participants to set targets, offer verbal rewards for completing their PA targets, and reminders of the coming PA programmes	“*Sometimes, I just forget to exercise* *I just don’t like to exercise, my grandson always calls me to encourage me to do some exercise, so I will walk around in the room.”*
	**Opportunity**				
** Group discussion**	**Motivation**	Environment restructuring modelling	3.1. Social support (unspecified) 6.2. Social comparison 12.2 Restructuring the social environment	Participants are encouraged to discuss their PA targets and behaviours within the group to seek peer support	*“You know, where I live, there’s no one else who speaks Chinese at my age. I really hope to find an exercise buddy so we can encourage each other. I think WeChat friends can be an alternative approach*”
	**Opportunity**				
**Other strategies**					
** Strength band**	**Opportunity**	Enablement	12.5. Adding objects to the environment	Participants will be offered a strength band to enable them to do strength and balance exercises at home	“*For them who cannot leave home to do exercise, you can teach them how to do exercise at home, for example, provide the strength band like this, it is very useful*.”
** Pedometer**	**Opportunity**	Enablement	2.3. Self-monitoring of behaviour 12.5. Adding objects to the environment	Participants will be offered a pedometer to enable them to self-monitor their PA behaviour	“*My smartphone can help me monitor how many steps I walk, and I check it often. I think it’s a good tool*.” “*Many of us received fitness trackers, but we don’t like using them. The screen is too small to read, and you have to charge it all the time.*”
	**Motivation**				
** Language support**	**Opportunity**	Enablement	3.2. Social support (practical) 12.2. Restructuring the social environment	All the information will be provided in accessible languages, including Traditional and Simplified Chinese	“*There is information available in English, but I cannot read it. There are local PA programs, but I’m not interested.”*
	**Capability**				

#### The booklet

The detailed content of the booklet is presented in Table 2. The developed booklet will include three parts: a culturally appropriate core booklet, the Chinese version of the Otago home exercise booklet, and a PA logbook. The information in the booklets was from reliable resources, including the UK Chief Medical Officers’ PA Guidelines [18], and the WHO PA guidelines [19]. The booklet also incorporates Chinese cultural factors. For example, it explains the importance of PA from the perspective of traditional Chinese medicine and philosophy and provides resources for traditional Chinese exercise programmes. A Chinese version exercise instruction booklet was translated based on the Otago home exercise booklet produced by Later Life Training [20]. Participants are also provided with a logbook to plan their PA, record daily PA, and track their achievements. All booklets are available in user-preferred languages, including Traditional and Simplified Chinese.

#### Workshop

Participants will be invited to attend three workshops during a 12-week period, scheduled every 4 weeks. Workshops will be designed to directly deliver the knowledge and information provided in the booklet. The workshop will include presentations, group exercises, and group discussions. When designing the content of the three workshops, we also considered the Transtheoretical Model (Stages of Change) [21]. This model has been used to design a walking group for older Chinese immigrants in the USA [22]. The workshops will progress from building awareness and capability (workshop 1), to planning and overcoming barriers (workshop 2), and maintaining PA and preventing relapse (workshop 3). A Chinese-speaking physiotherapist will be invited to demonstrate strength and balance exercises from the Otago home exercise programme [20, 23]. The workshop also serves as an avenue for older Chinese adults to connect, find exercise partners, and cultivate peer support before they are invited to the social media group. Each workshop will last about 90 min.

#### Social media group

Participants will be invited to join a study special WeChat group (WeChat is a popular social media platform among the Chinese population) or WhatsApp group as an online peer support platform for participants to share their PA targets and progress and encourage each other, and also receive motivational messages and reminders about upcoming PA programmes from the researcher. Participants can choose to use the social media platform they are familiar with, either WeChat or WhatsApp.

In addition, participants will receive a resistance band to complete the strength exercises from the Otago programme, and a pedometer, which serves as both an intervention component by supporting behaviour monitoring and goal setting, and a tool for data collection.

### Control group (WHO leaflet group)

In the control group, participants will receive the printed leaflet of recommendations from the WHO on PA for older adults [19]. Participants will be encouraged to follow these guidelines, 150–300 min of moderate or 75–150 min of vigorous aerobic activity per week, along with balance and strength exercises twice weekly, at a time and place convenient to them. No additional support will be provided. Participants in the control group will also be provided with pedometers to collect their steps at the three time points.

The study will span 18 weeks, comprising a 12-week intervention phase and a 6-week follow-up phase. During the intervention phase, participants in the intervention group will attend workshops and receive motivational and reminder messages via a social media group. In the follow-up phase, they will be encouraged to continue their PA behaviour independently without further workshops or messages. Participants in the control group will be encouraged to follow the WHO PA guidelines during the 18-week study period and, after the study concludes, will receive the culturally appropriate PA booklet and be invited to attend one workshop.

### Data collection

Baseline screening and measurements will be conducted for all eligible participants before randomisation (T1). Subsequently, measurements will be taken at two additional time points: post-intervention (T2, 12 weeks after randomisation) and after the follow-up period (T3, 18 weeks after randomisation). A summary of the schedule of enrolment, interventions, and assessments is presented in Table 3.

**Table 3:**
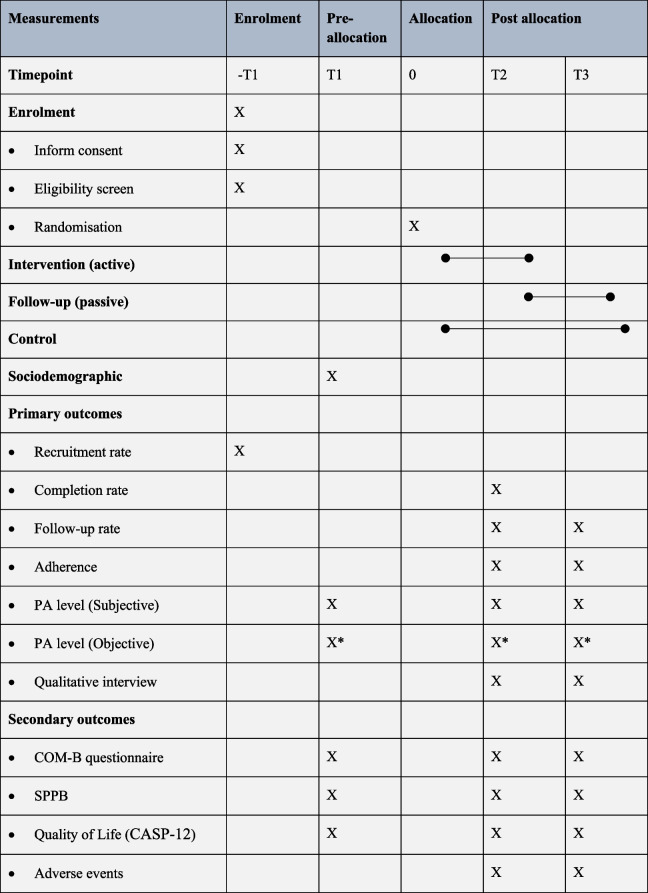
Schedule of enrolment, interventions and assessments

*Abbreviation:* *PA* physical activity, *COM-B* Capability, Opportunity, Motivation, and Behaviour, *SPPB* Short Physical Performance Battery test, *CASP-12* Control, Autonomy, Self-realisation, and Pleasure-12 *Participants’ physical activity was monitored for 7 consecutive days from this point

#### Sociodemographic

Sociodemographic information will be collected at baseline. The data collection table will be constructed based on the suggestions of PROGRESS plus [24] and information about participants’ migrant status and language proficiency. The following information will be collected, including, for example, age, gender, employment status, years of education, language spoken at home, fluency in English, years of residence in the UK, medical history and fall history within the past 6 months, and current PA level. We will also collect information on participants’ general practice (GP) details so that in the event of any adverse effects, we can report these to their GP promptly when necessary.

#### Primary outcomes

##### Feasibility outcomes

Recruitment rate, compliance rate, adherence, and follow-up rates will be monitored throughout the entire study, including the recruitment, screening, intervention, and follow-up periods.

##### Recruitment rate

We aim to recruit at least 30 participants within 8 weeks. Detailed records will be kept of the total number of potential participants approached by the researcher, the number of participants who attend the screening, and the number of participants who are ultimately included in the study.

##### Retention rate

Randomised participants may exit the trial for various reasons. A research journal and case report form will be created to record the number of participants who (1) complete all the follow-up data collection; (2) fully withdraw, where participants refuse to engage in any future activities (including intervention and assessment); (3) loss to follow-up, which should only be considered after the 12-week and/or 18-week visits are due and all contact attempts have been exhausted, but still cannot complete the data collection; and (4) death or other serious adverse events. It is recommended not to automatically treat all withdrawals as a complete exit from the trial [25]. Participants should not be fully withdrawn for discontinuing or noncompliance with the intervention. For example, participants may choose to withdraw from attending the workshop or participating in the social media group, but may still be willing to engage in data collection activities. Alternatively, their participation may need to be discontinued based on clinical judgement. We will make every effort to collect as much data as possible to maintain the integrity of the study. The reasons for participants’ exit from the trial will be carefully recorded separately.

##### Adherence

Adherence to the PA protocol will be determined based on the type of intervention [26]. In this study, adherence will be mainly assessed by the number of workshops attended by the participants.

##### Qualitative interviews

At the end of the study, about 50% of participants (including those who withdraw from the trial) will be invited for follow-up interviews. The majority of participants will be from the intervention group. We aim to recruit participants with different adherence levels to gain a comprehensive understanding of their experiences. We will also invite a small number of participants from the control group to understand their experience of being randomised to the control group and how the WHO PA booklet influences their PA levels.

The interview aims to explore older Chinese adults’ experiences of participating in the trial, their overall perceptions of the PA promotion strategy package, and the potential impact the intervention may have had on their PA behaviour. The interview topics will be developed based on the guidance suggested by O’Cathain et al. in 2015. The following topics will be discussed: (1) the recruitment and randomisation process; (2) the PA promotion intervention and ideas for improvement of the intervention; (3) the outcomes and measurements [27].

##### PA behaviour outcomes (T1, T2, and T3)

PA level will be measured three times, at the baseline, after the intervention and after the follow-up. PA, being a multi-dimensional construct, presents challenges when attempting to measure all its facets using a single method. As recommended by Sylvia et al. in 2014, this study will employ a combination of objective and subjective methods to assess participants’ PA levels before and after the intervention [28]. Considering the potential discrepancies, we will prioritise the objective pedometer scores. However, qualitative interviews will be conducted to explore and explain the underlying causes of discrepancies.

##### Subjective measures of PA

Subjective measurement will be conducted using the Chinese version of the Physical Activity Scale for the Elderly (PASE). PASE is a brief and easily scored survey designed specifically to assess the PA of persons aged 65 years and older [29].

Numerous self-reported PA questionnaires have been documented in the literature. A recent systematic review published in 2020 analysed the measurement properties of 40 self-administered questionnaires that assess PA in older adults. This review suggested that PASE demonstrated sufficient reliability and validity, making it a suitable choice to assess total PA in this study [30]. In addition, it is worth noting that a Chinese version of the PASE questionnaire was published in 2012 [31]. This further strengthens the appropriateness of using the PASE questionnaire as a measurement tool for assessing PA in this study.

##### Objective measures of PA

Objective measurement of PA can be measured by accelerometers, pedometers, or other wearable devices [32, 33]. This approach aims to address the limitations associated with subjective self-report measures, including recall bias, social desirability bias, and inaccuracies in estimating activity intensity and duration. By utilising the objective measurement methods, a more accurate and reliable quantification of PA can be achieved, providing an accurate assessment of participants’ actual changes in PA levels before and after the intervention.

Ideally, accelerometers would be used to ensure accurate and comprehensive objective PA data, including PA duration and intensity. However, due to limited resources in this project, a pedometer (Realalt 3DTriSport 3D Pedometer; 3DTriSport Pedometer for Walking with Clip and Lanyard – Realalt) will be used to monitor participants’ PA level (i.e. step counts). The pedometer was chosen for its accuracy and affordability, and it has been previously used to measure preoperative PA levels in patients undergoing anatomical lung resection [34]. In addition, the Realalt 3DTriSport 3D Pedometer has 30 days memory, which offers an advantage by reducing the participant’s burden of daily step logging and minimising self-reporting errors. Following each of the three measurement points (T1, T2, T3), participants’ PA will be monitored continuously for 7 consecutive days using the pedometers.

#### Secondary outcomes

The following secondary outcomes will be measured at three time points: T1, T2, and T3.

##### COM-B questionnaires

Based on the COM-B framework, the PA promotion strategies in this study aim to improve older Chinese adults’ capability, motivation, and opportunity for PA, making it essential to assess changes in these perceptions before and after the intervention.

Brief COM-B questionnaires will be used to assess the perceived capability, opportunity, and motivation for PA behaviour among older Chinese adults in the UK. The acceptability, reliability, and validity of this brief measurement tool have been tested by Keyworth [35]. The questionnaires have been adapted to understand behaviour, such as hand hygiene behaviour among hospital staff [36], and PA among adults in the UK during the COVID-19 pandemic [37]. The questions have been adapted based on the findings of the interview with the target population. Example questions relate to opportunities like “There are suitable places and programs near me for PA, such as parks, swimming pools, or opportunities for dancing and Tai Chi”. The social environment is supportive of PA, such as exercise groups, encouragement from family and friends, and sufficient time to be active. Participants responded on a scale of 0 (strongly disagree) to 10 (strongly agree). Both total and domain-specific analyses of the COM-B questionnaire will be conducted to assess overall and domain-level changes in capability (physical and psychological), opportunity (physical and social), and motivation (reflective and automatic) for engaging in PA.

##### Physical function

The Short Physical Performance Battery (SPPB) will be used to assess the potential change of physical function before and after the PA promotion strategies [38]. This is an objective tool used to measure balance, lower extremity strength, and functional capacity in older adults aged 65 and above [39]. The test assesses functional mobility across three domains: walking, sit-to-stand, and balance. Additionally, the SPPB is a brief assessment, designed to minimise participant burden.

##### Quality of life

The CASP-12 (Control, Autonomy, Self-realisation, and Pleasure-12) questionnaire will be used to measure older adults’ health-related quality of life [40]. This questionnaire consists of 12 items, each rated on a scale from 1 (often) to 4 (never). The items are summed to calculate a total score. CASP-12 has recently been tested among older Chinese migrants in New Zealand, which shows evidence for the validity and reliability of the CASP-12 as a QOL measure to be used with older Chinese migrants [41].

##### Adverse events

A safety reporting protocol will be followed for managing related and unexpected serious adverse events (SAEs) and directly attributable adverse events (AEs). All AEs will be documented in the Case Report Form (CRF). Participants will be asked to inform the researcher of any injuries after seeking appropriate medical assistance. They could stop the exercise at any time if they experienced any adverse. If a serious adverse event (AE) occurs, it will be reported to the research team to assess whether further reporting to the trial sponsor and ethics committee is required, in line with the safety reporting protocol.

### Data analysis

This study will generate both quantitative data and qualitative interview data.

Quantitative data will be analysed using SPSS software [42]. Data will be checked for completeness and accuracy prior to analysis. Descriptive statistics will summarise participant characteristics, feasibility outcomes, and study measures. Continuous variables will be reported as means and standard deviations (SD) or medians and interquartile ranges (IQR), depending on data distribution; categorical variables will be summarised as frequencies and percentages. Feasibility outcomes, including recruitment, retention, adherence, fidelity, and data completeness, will be reported descriptively.

Exploratory effectiveness outcomes will be summarised by reporting means and SDs for each group at baseline and post-intervention, with 95% confidence intervals (CIs) calculated to estimate changes following the intervention. Analyses will follow an intention-to-treat principle. As this is a feasibility study, no formal hypothesis testing or between-group comparisons will be conducted.

The extent of missing data will be reported descriptively as part of the feasibility outcomes. Qualitative data will be collected to explore reasons for missing data and inform strategies to minimise missingness in future studies.

The interview data will be analysed using the NVivo software. The thematic analysis method will be employed [43]. This analysis aims to understand participants’ views and acceptability of the PA intervention and the trial process. The interview data will be coded by the lead researcher (YY) and discussed with the research team.

### Public and community involvement and engagement

A project advisory group (PAG) was established for this project to ensure that the intervention and trial are both meaningful and acceptable to older Chinese adults in the UK. The PAG consists of four PCIE representatives: two leaders of Chinese associations in Manchester, both with nursing backgrounds; a Chinese-speaking physiotherapist; and an older Chinese adult with an interest in PA, all of whom have experience working closely with older Chinese adults in the UK. The PAG group were consulted to ensure the strategies were appropriate. The group contributed to the design of the intervention, the determination of inclusion and exclusion criteria, outcome assessments, progression criteria, and participant recruitment.

### Ethics issue

This study has been reviewed and approved by the University of Manchester Research Ethics Committee 2 (Ref: 2024–19749-37,005). The research will comply with the principles of Good Clinical Practice and will follow all research laws and regulations within England. Participation in this study is entirely voluntary. Enrolled participants will be informed that they can withdraw from the study at any time.

### Intervention fidelity

Fidelity refers to how closely the delivered intervention matches the originally planned or designed protocol [44]. In this study, intervention fidelity reflects the extent to which the three main components of the intervention are delivered as planned. A fidelity checklist will be used to systematically assess these components and will be completed by the lead researcher. The checklist can be found in Appendix 2. Specifically, fidelity is assessed by checking:

Whether all participants in the intervention group receive the culturally tailored PA promotion booklets; Whether the three-monthly workshops are organised and delivered according to the planned time and content; Whether at least three motivational messages are sent to participants via the social media support group as intended each week. Additionally, fidelity monitoring includes recording whether all participants receive a pedometer and resistance band to support home-based exercise.

### Data monitoring and quality assurance

We are collecting and storing personal information in line with the General Data Protection Regulation (GDPR) and the Data Protection Act 2018. A data management plan has been established to ensure that all data is properly documented and securely stored. The lead researcher will be responsible for recruiting all participants, randomising them to intervention or control and collecting research data. All research data will be stored in a secure folder on password-protected, encrypted computers. Access to the data collected during the study is available only to the research team. The key linking real names to pseudonymised data will be stored separately in an encrypted file accessible only to the lead researcher. Pseudonymised data and transcripts will be stored for 5 years following the end of the study.

### Progression criteria

Progression criteria for a full RCT are developed following the guidance of Avery et al. [45]. The thresholds are informed by findings from earlier reflective research [46, 47], internal team discussions, and consultation with the PCIE group. In line with Mellor et al. (2023), we define progression criteria across multiple trial domains, including recruitment, retention, data completeness, fidelity, safety, and intervention acceptability, drawing on both qualitative and quantitative data [48]. Full details of the progression criteria are provided in Table 4.

**Table 4:** Progression criteria

Domain	Go-proceed with RCT	Amend-proceed with change	Stop-do not proceed
Recruitment	If at least 30 participants are recruited within eight weeks	If at least 16–29 participants are recruited within eight weeks	If ≤ 15 participants are recruited within eight weeks
Retention	If ≥ 24 participants (80%) complete the 18-week follow-up	If 20–23 participants (64–79%) complete the 18-week follow-up	If ≤ 19 participants (63%) complete the 18-week follow-up
Adherence	If an average of ≥ 12 of the 15 participants (80%) attend each workshop	If an average of 8–11 of the 15 participants (53–73%) attend each workshop	If an average of ≤ 7 of the 15 participants (46%) attend each workshop
Data completeness	If ≥ 90% of the data is completed	If 70–89% of the data is completed	If ≤ 69% of the data is completed
Fidelity	If all core intervention components are delivered as planned	If minor deviations in the delivery of core components	If major deviations in the delivery of core components
Safety	No serious adverse events; minor events, if any, are manageable and anticipated	Some minor adverse events; no serious harm, but may need adjustments	Serious adverse event(s) related to the intervention were reported
Acceptability	Qualitative data indicate strong acceptability of the intervention components	Qualitative data indicate general acceptability, with changes needed	Qualitative data indicate the intervention was unacceptable

## Discussion

This is the first trial known to address physical inactivity among older Chinese adults in the UK, a group often overlooked in mainstream health interventions. Although RCTs are considered the gold standard for assessing the effectiveness of health interventions, it is strongly recommended to conduct a pilot and feasibility study before a full-size RCT [49]. A feasibility RCT ensures that a larger trial will be scientifically sound, ethically justified, and resource-efficient [50, 51]. A feasibility study can also provide information for intervention modification to make it more acceptable to the target population [52].

Progression criteria are developed to assess feasibility, guide trial and intervention modifications, and support decision-making about progressing to a definitive trial. There are no universally agreed-upon criteria or thresholds in the literature, as a single standard is unlikely to suit all studies. It is therefore important to consider the specific context of each trial [48]. For example, Hynes et al. [46] used the successful recruitment of all 20 patients with multimorbidity in primary care (100%) as a criterion for progressing to an RCT, while Young et al. [47] proposed a 50% recruitment rate among people living with frailty and receiving haemodialysis as the threshold for a “go” decision.

The progression criteria in this study are shaped by the characteristics and context of the target population. Although ethnic minority groups, including older Chinese adults in the UK, are often described as hard to reach, the research team has built trust with this community through sustained engagement and previous research activities, which helps ensure successful recruitment. The study specifically targets older Chinese adults who do not currently meet recommended PA guidelines. While eligibility criteria were initially considered as part of the progression plan, findings from earlier research and feedback from the PCIE group confirmed that most older Chinese adults in the UK are physically inactive. This makes the eligibility criteria easily achievable and therefore less critical as a progression indicator for this feasibility study. In addition, compared with traditional clinical trials focusing on medical interventions, behaviour change interventions such as PA promotion are viewed by the PCIE group as more acceptable. This is likely to further support recruitment, retention, acceptability, and safety. Taken together, this context supports the use of relatively strict recruitment criteria, aiming to recruit 100% of the intended sample within the planned timeframe to ensure the feasibility of a future full RCT. More flexible thresholds for retention and adherence allow for appropriate modifications, consistent with the overall objectives of the study. 

The intervention in this trial was developed through in-depth interviews with the target population to ensure its alignment with their cultural values, preferences, and specific barriers to engaging in PA [53]. Utilising the Behaviour Change Wheel, a series of BCTs were identified to enhance participants’ capability, opportunity, and motivation to engage in PA [9, 54]. These techniques were implemented through a package comprising booklets, three workshops, and a social media support group, all tailored to the cultural context of older Chinese adults.

These components were chosen to provide a multi-faceted approach that combines traditional educational materials with interactive and peer-supported elements. The booklets serve as a tangible resource for participants, enabling them to engage with the content at their own pace. This comprehensive package comprises three components: the culturally appropriate core booklet, the Chinese version of the Otago exercise booklet, and the PA logbook. Each page of the core booklet was thoughtfully designed to address capability, opportunity, and motivation-related barriers to PA among older Chinese adults, as summarised in Table 3. Recognising that many participants are unfamiliar with how to perform strength and balance exercises, the Otago exercise booklet, an evidence-based programme proven to prevent falls [55], was translated into Chinese to meet the needs of older Chinese adults in the UK. In addition, a PA logbook was designed to help participants set PA targets, create activity plans, and sustain their PA behaviours. This decision was based on participants’ reported need, as many indicated that PA is not part of their routine. It also aligns with evidence suggesting that goals and planning are effective BCTs for promoting PA among older adults, fostering both motivation and achievement [56].

The social media group is intended to foster ongoing support and engagement. As an ethnic minority group, older Chinese adults in the UK often live in dispersed areas, making it challenging to find exercise buddies in their local communities. Virtual peer support provides an alternative option to address this barrier. It also acknowledges the growing adoption of technology among older adults [57]. Instead of using a single type of social media, as suggested by PCIE activities, participants can choose between WeChat and WhatsApp to enhance the acceptability of the intervention component. Both types of social media have been used to deliver PA interventions in previous studies and have been shown to be well accepted by older adults [58, 59]. This approach could provide valuable evidence for determining the most suitable social media support method for this population in further studies.

### Practical consideration

The study aims to recruit at least 30 participants within a timeframe of 8 weeks. Collaborative efforts have been made to enhance recruitment, including securing support from the Chinese associations. A qualified Chinese-speaking physiotherapist has agreed to lead the group Otago exercises. Additionally, the university will provide a venue for the workshops. By addressing these practical considerations, the study ensures adherence to ethical guidelines, establishes effective recruitment strategies, secures suitable intervention venues, and considers incentives to boost participant engagement.

### Limitations and challenges

The study’s small sample size is a limitation inherent to feasibility studies, which could affect the generalisability of the findings. However, our primary aim is to test the intervention’s feasibility, and the insights gained will be crucial for planning a larger trial. Convenience sampling will be employed in feasibility studies due to its practicality and ease of participant recruitment. This approach may introduce potential bias and limit the generalisability of the findings. To address this limitation and enhance the applicability of the study’s results, we will recruit participants from a wide range of demographics, including diverse genders, ages, language backgrounds, and PA levels. By actively engaging individuals with varied characteristics and activity levels, we aim to gain a comprehensive understanding of the intervention’s feasibility across different subgroups within the target population [60]. Additionally, as with all self-report measures, responses on instruments such as the PASE and COM-B questionnaires may be subject to social desirability bias, potentially influencing the accuracy of reported behaviours and perceptions [61].

### Implications for full-scale RCT

If the progression criteria are met, the study will pave the way for a larger RCT that could have significant public health implications. The results will also inform necessary adjustments to the intervention, such as refining the booklet content, optimising workshop delivery, or enhancing the social media group’s functionality. While this intervention was developed within the UK context, its culturally tailored approach may offer valuable insights for adaptation and scalability in other countries with growing older Chinese populations or other ethnic communities, where cultural sensitivity is key to success.

## Conclusion

This feasibility RCT is a crucial step towards understanding how to effectively promote PA among older Chinese adults in the UK. By grounding the intervention in the lived experiences and cultural contexts of the target population, this study not only tests the feasibility of the proposed strategies but also sets the stage for future research that could lead to meaningful health improvements in this underserved group. The findings from this study will guide the development of a full-scale RCT, with the ultimate goal of creating culturally appropriate, sustainable interventions that improve the health and well-being of older Chinese adults in the UK.

## Trial status

As of the time of manuscript submission, participant recruitment for this study was still in progress.

## Supplementary Information

Supplementary Material 1.

Supplementary Material 2.

Supplementary Material 3.

## Data Availability

The datasets used and/or analysed during the current study are available from the corresponding author on reasonable request.

## Abbreviations

AE: Adverse event
BCT: Behaviour change technique
COM-B: Capability, Opportunity, Motivation, and Behaviour
CASP-12: Control, Autonomy, Self-realisation, and Pleasure-12
CI: Confidence intervals
GDPR: General Data Protection Regulation
PAG: Project advisory group
PCIE: Public and community involvement and engagement
RCT: Randomised controlled trial
SPPB: Short Physical Performance Battery Test
SD: Standard deviations
UK: United Kingdom
PIS: Participant information sheet
PASE: Physical Activity Scale for the Elderly
