# Supplementary material for: Culturally appropriate physical activity promotion strategy package among older Chinese adults in the UK: a feasibility randomised control trial protocol
Source: Pilot Feasibility Stud. 2025 Nov 5;11:134. doi: 10.1186/s40814-025-01693-7 (PMC12587680; doi:10.1186/s40814-025-01693-7)
Supplement: Supplementary file 1 — Supplementary Material 1. [file 40814_2025_1693_MOESM1_ESM.pdf]

A Feasibility Study: Culturally Sensitive Exercise Promotion Strategies for Chinese Seniors in the UK

针对在英国定居的华人长者的文化特色的运动促进策略：

可行性研究

Consent Form

知情同意书

If you are happy to participate please complete and sign the consent form below.

如果您愿意参加本研究，请填写并签署以下同意书。

	**Activities**<br><br>**活动**	**Initials**<br>**姓名首字母**
1	I confirm that I have read the attached information sheet (Version 3, Date 09/08/2024) for the above study and have had the opportunity to consider the information and ask questions and had these answered satisfactorily.<br><br>我确认我已经阅读了所附的关于上述研究的信息表(版本 3，日期 2024 年 8 月 9 号)，有机会考虑这些信息和提出疑问，并得到了令我满意的回答。	
2	I agree to answer the screening questions related to my physical activity level and health condition to determine if I am eligible to take part in this study.<br>我同意回答与我的身体活动量和健康状况相关的筛选问题，以确定我是否符合参加此研究的资格。	
3	I understand that my participation in the study is voluntary and that I am free to withdraw at any time without giving a reason and without detriment to myself. I understand that it may not be possible to remove my data from the project once it has been pseudonymised and forms part of the data set<br>我理解我参与本研究是自愿的，我可以随时退出而无需提供理由，也不会对自己造成任何不利影响。我理解，一旦我的资料被去识别化并成为资料集的一部分，可能无法从专案中删除我的资料。<br>I agree to take part on this basis.<br>在此基础之上我同意参加本研究。	

4	I agree that any data collected may be included in anonymous form in publications/conference presentations.<br>我同意所收集的资料可能会以匿名的形式出现在出版物/会议报告中。	
5	I understand that data collected during the study may be looked at by individuals from The University of Manchester or regulatory authorities, where it is relevant to my taking part in this research. I give permission for theseave to my taking part in this research. I give permission for theseave tos unity for theseave s.<br>我明白来自曼彻斯特大学或监管机构的个人可能会查看该研究所收集的数据，这可能涉及我所提供的数据。 我允许这些人存取我的资料。	
6	I understand that there may be instances where during the course of the research information is revealed which means the researchers will be obliged to break confidentiality and this has been explained in more detail in the information sheet.<br>我了解在研究过程中可能会出现资讯外泄的情况，在某些情况下研究人员将需要违反保密协议，这些资讯已经在邀请函中进行了更详细的解释。	
7	I agree to take part in this study.<br>我同意参加这项研究。	

The following activities are optional, you may participate in the research without agreeing to the following:
以下活动是可选的，您可以在不同意以下内容的情况下参与研究：

8	I agree that the research team may retain personal data, including screening information, regardless of whether I am eligible or not. This will be securely stored for up to two years in compliance with the Data Protection Act.<br>我同意研究团队可以保留我的个人数据，包括筛查信息，无论我是否符合参与条件。这些数据将根据《数据保护法》安全存储，最长保存两年。	
9	I agree to be contacted and take part in any follow-up interview.<br>我同意研究人员将来可能会联系我进行后续访谈。	
10	I agree to receive a gift voucher for each assessment I complete, and I understand my full name will be passed to the University's Finance team for audit purposes.<br>我同意接受每次评估后获得的礼券，并且我明白我的全名将会提交给大学的财务部门以供审计使用。	
11	I agree that any anonymised data collected may be made available to other researchers	

	我同意可以将收集到的任何匿名资料提供给其他研究人员	
12	I agree that the researchers may retain my contact details in order to provide me with a summary of the findings for this study.<br>我同意研究人员可以保留我的联络方式，以便向我提供研究结果摘要。	

Data Protection

资料保护

The personal information we collect and use to conduct this research will be processed in accordance with UK data protection law as explained in the Participant Information Sheet and the Privacy Notice for R esearch Participants .

我们收集和用于进行这项研究的个人资讯将根据英国资料保护法进行处理，如参与者资讯表和研究参与者隐私权声明中所述。

________________________ ________________________ _____________

Name of Participant Signature Date

参与者姓名 签名 日期

________________________ ________________________ _____________

Name of the person taking consent Signature Date

研究者姓名签名日期

[One copy of the consent will be left with the participant and the main researcher will keep one copy for the record of this research]

[同意书一份留给参与者，主要研究者保留一份作为本次研究的记录]
