# Supplementary material for: Culturally appropriate physical activity promotion strategy package among older Chinese adults in the UK: a feasibility randomised control trial protocol
Source: Pilot Feasibility Stud. 2025 Nov 5;11:134. doi: 10.1186/s40814-025-01693-7 (PMC12587680; doi:10.1186/s40814-025-01693-7)
Supplement: Supplementary file 2 — Supplementary Material 2. [file 40814_2025_1693_MOESM2_ESM.docx]

**Appendix 2. The intervention fidelity checklist**

Title of Study: Culturally sensitive physical activity promotion strategy package among older Chinese adults in the UK: A feasibility randomised control trial protocol

| **Intervention component** | **Record** |
| --- | --- |
| **Number of participants who received all three booklets** |  |
| **Number of participants who received the pedometer** |  |
| **Number of participants who received the Strength band** |  |
| **Monthly workshops** |  |
| **Workshop 1** |  |
| - Education Presentation – PA guideline |  |
| - Group Otago exercise delivered by the Chinese-speaking physiotherapist |  |
| - Group discussion |  |
| **Workshop 2** |  |
| - Education Presentation – barriers and facilitators |  |
| - Group Otago exercise delivered by the Chinese-speaking physiotherapist |  |
| - Group discussion |  |
| **Workshop 3** |  |
| - Presentation – the perceived benefit of PA |  |
| - Group Otago exercise delivered by the Chinese-speaking physiotherapist |  |
| - Group discussion |  |
| **Motivational message from the social media group** |  |
| - No. of motivational messages sent in Week One |  |
| - No. of motivational messages sent in Week Two |  |
| - No. of motivational messages sent in Week Three |  |
| - No. of motivational messages sent in Week Four |  |
| - No. of motivational messages sent in Week Five |  |
| - No. of motivational messages sent in Week Six |  |
| - No. of motivational messages sent in Week Seven |  |
| - No. of motivational messages sent in Week Eight |  |
